# Combined Germline and Mosaic SDHA Mutation Is Associated With a Multicancer Syndrome Including Neuroblastoma, Renal Cancer, and Multifocal GI Tumor

**DOI:** 10.1200/PO.23.00455

**Published:** 2024-06-17

**Authors:** Lee D. Cranmer, Eric Q. Konnick, Jennifer R. Yoshida, Angela L. Jacobson, Bilal A. Malik, Harveshp Mogal, Lucas B. Sullivan, Cynthia L. Handfrod, Colin C. Pritchard, Marianne E. Dubard-Gault

**Affiliations:** ^1^ Department of Medicine, University of Washington, Seattle, WA; ^2^ Department of Laboratory Medicine and Pathology, University of Washington, Seattle, WA; ^3^ Fred Hutchinson Cancer Center, Seattle, WA; ^4^ Division of Nephrology, Department of Medicine, University of Washington, Seattle, WA; ^5^ Department of Surgery, University of Washington School of Medicine, Seattle, WA; ^6^ Human Biology Division, Fred Hutchinson Cancer Center, Seattle, WA; ^7^ Department of Laboratory Medicine and Pathology, University of Washington, Seattle, WA; ^8^ Fred Hutch Cancer Center, Seattle, WA; ^9^ Swedish Cancer Institute and the Paul G Alle Research Center, Seattle, WA; ^10^ Translational Science and Therapeutics Division at the Fred Hutchinson Cancer Center, Seattle, WA

## Abstract

Highlighting here a patient case with neuroblastoma, renal cancer & GIST from germline SDHA.

## Introduction

GI stromal tumors (GISTs) are the most common mesenchymal tumors of the GI tract. They arise from the interstitial cells of Cajal within the myenteric plexus. The mainstay of treatment is surgical resection followed by targeted therapy. Succinate dehydrogenase (SDH) protein–deficient GISTs often retain IHC expression of CD117 (c-Kit).^1^ Evaluation of SDH protein deficiency or mutations is critical in *KIT/PDGFRA*-negative GISTs as SDH-deficient GISTs are less responsive to imatinib (Gleevec) and 70% of these patients develop distant metastases.^2-6^ Hereditary paraganglioma and pheochromocytoma syndrome are rare genetic conditions associated with pathogenic variants in the SDH complex (*SDH(x)*) genes. This hereditary cancer syndrome (HCS) is also called familial paraganglioma and pheochromocytoma syndrome (familial PGL/PCC). Patients with familial PGL/PCC are at an increased risk of developing paragangliomas, pituitary adenomas, pheochromocytoma, GISTs, and renal cell cancers. Identification of familial PGL/PCC matters for immediate surgical risk as patients with an occult pheochromocytoma can develop a catecholamine storm with uncontrolled tachycardia, hypertension, and cardiac arrest. Identification of this HCS also guides family testing, screening, early detection, and preventative interventions.

## Case Scenario

**FIG 1.**
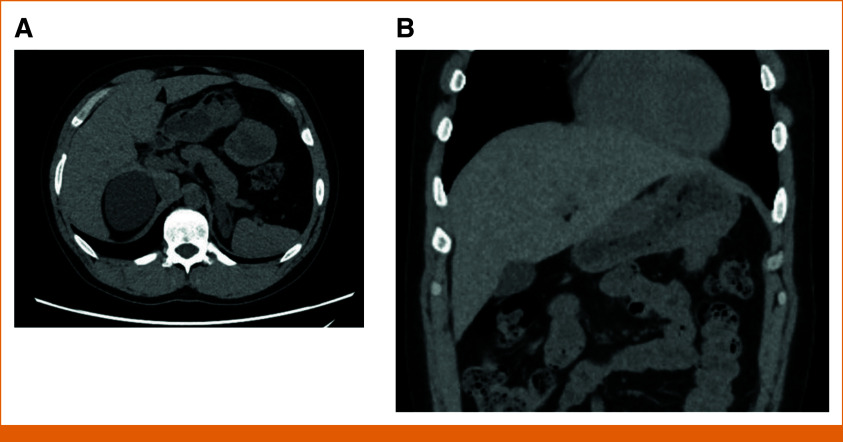
Abdominal imaging of this patient. (A) Large mass from GIST in the cross-sectional view. (B) GIST and stomach lining thickening in the coronal view. GIST, GI stromal tumor.

**FIG 2.**
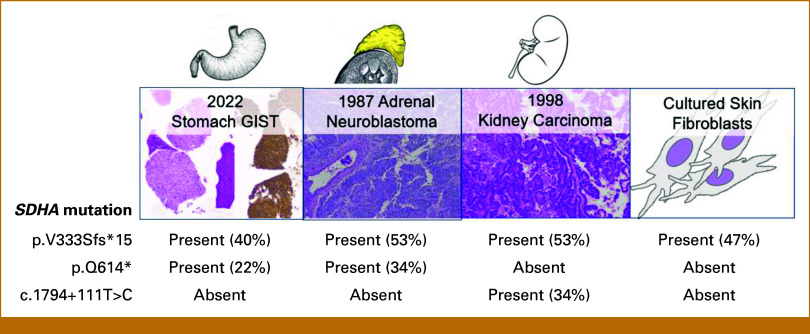
Tumors from this patient with variant allele fractions for *SDHA* mutations. Top: first panel: GIST biopsy, H&E stain at ×5 and c-KIT IHC stain at ×5. Second panel: neuroblastoma H&E stain at ×5. Third panel: renal cell carcinoma, H&E stain, ×5. Last panel: drawing of cultured fibroblasts. Bottom: SDHA mutations. GIST, GI stromal tumor; H&E, hematoxylin and eosin.

Our patient initially presented with abdominal symptoms at 3 years in 1986. He was diagnosed with a stage III right adrenal neuroblastoma with unfavorable Shimada classification treated with surgical resection. He developed recurrence in his chest and abdomen in 1987, and he received sequential regimens containing doxorubicin, cisplatin, dacarbazine, cyclophosphamide, and etoposide. He was then conditioned with cyclophosphamide and melphalan and received total body irradiation of 1,200 cGy. He underwent matched related donor bone marrow transplant from the fraternal twin brother and achieved remission. He was on cyclosporine and methotrexate for graft-versus-host disease for 2.5 years. A left exophytic renal lesion suspicious for renal cell cancer was identified on surveillance scans. He underwent radical left nephrectomy at 15 years in 1998 and developed chronic kidney disease complicated by gouty arthritis. His creatinine was 1.4-1.8 mg/mL with eGFR in 40 ml/min for a decade before progression. In 2017, he had a symptomatic right thigh schwannoma removed. After an abrupt seizure in 2019, he was diagnosed with an atypical meningioma, WHO grade II, and seizures resolved after surgical resection. During workup for kidney transplantation in 2020, he had multiple incidental exophytic gastric masses on computed tomography (CT), largest measuring 4.7 × 5.5 × 6.1 cm, 1-cm segment three hepatic lesions, a 2.8-cm right adrenal nodule, a 6.4 × 5.8-cm left renal cyst, and a 1.4-cm right thyroid nodule (Fig 1). He had no pulmonary chondromas. Samples from endoscopic ultrasound redemonstrated multiple subepithelial masses in the stomach. Pathology was consistent with GIST with immunohistochemistry staining strongly and diffusely positive CD117 (c-KIT) and DOG1 and negatively for S100 calcium-binding protein. Necrosis was present with one mitotic figure in the 15 high-power field. His GIST sample was tested using the UW-OncoPlex next-generation sequencing^7^ test. The University of Washington allowed performing a deep-dive genetic analysis. Given cancer history, he was referred to our clinical cancer genetics service. On physical examination, the height was 177 cm and the weight was 69 kg for a BMI of 23.5 kg/m^2^. He had freckles throughout, no café au lait macules, no head and neck tumors, and no lymphadenopathy, abdomen was diffusely tender, but soft palpation and musculoskeletal and neurologic examination were normal. Serum plasma metanephrines were normal. There were no vestibular schwannomas on previous brain magnetic resonance imaging (MRI). Germline genetic testing was sent on cultured fibroblasts. The UW laboratory obtained specimens from 1987, 1998, 2001, and 2021. GIST was negative for mutations in exons 9, 11, 13, and 17 of *KIT* and in exons 12 and 18 of *PDGFRA*. Our patient was diagnosed with familial paraganglioma and pheochromocytoma syndrome (familial PGL/PCC). Tumor profiling and germline genetic testing results for the gene *SDHA* (NM_004168.4) are summarized in Figure 2. All three samples and DNA from the cultured fibroblast were run on the same OncoPlex cell. Data were analyzed looking for evidence of mosaicism. Several copy number changes were detected in the GIST without a clear overlap with neuroblastoma from 1987. Neuroblastoma tumor had many additional somatic mutations and copy number changes, with none overlapping with the GIST tumor mutation profile from 2021. Kidney carcinoma had a candidate second somatic hit in a deep intronic region called c.1794=111T>C at 34% variant allele fraction (VAF) and a pathogenic variant in the gene *SDHD* (NM_003002.4): c.279C>A (p.Y93*) at 32% VAF. Somatic mutations in *NF2* and *CDKN1C* were also detected in the kidney carcinoma without an overlap with any other tumors sequenced. Consensus in multidisciplinary conference was to start dose-adjusted sunitinib (Sutent) at 25 mg per day for 4 weeks on/2 weeks off schedule over full-dose regorafenib after pharmacy's input given limited knowledge or data for patients with end-stage renal disease. He kept a strict kidney diet, and he tolerated sunitinib well. The dose was increased to 50 mg once per day after 6 months. Patient consented to participate in our repository and registry. Patient had stable to slight regression of disease on repeat CT of abdomen at 6, 9, and 12 months, making him eligible for gastrectomy at 15 months of therapy. A fresh frozen surgical specimen was sent to the laboratory to attempt developing a patient-derived xenograft model. Patient’s first upper extremity arteriovenous (AV) fistula did’ not mature appropriately, and he was scheduled to receive another AV fistula further out from Sutent exposure. After recovery, he was scheduled for screening colonoscopy and found to have three 30-mm tubular adenomas and more than 25 sessile polyps. He will continue to receive colonoscopy every 6 months.

## Discussion

This is the second reported case linking a neuroblastoma tumor with an inherited germline genetic diagnosis of *SDHA*-familial PGL/PCC.^8^ After 3 separate primary cancers, germline genetic testing results brought a unifying diagnosis to the questions this patient had for decades. *SDHA* pathogenic variant called c.996del (p.V333Sfs*15) was present across all his samples at a VAF close to 50%, meaning that this variant was either inherited or arose in utero during early embryonic development.^9^ Peripheral blood was not tested as the patient was a bone marrow recipient from his fraternal twin brother. We have offered family testing to unaffected parents to help confirm de novo inheritance above. Outstandingly, we identified the same second *SDHA* pathogenic variant c.1840C>T (p.Q614*) in more than one neoplasm, the neuroblastoma, and the GIST diagnosed 34 years apart. Two hypotheses were raised as explanations, either (1) a second postzygotic mutation in *SDHA* existed in this patient, where the same mutation arose independently in multiple neoplasms, or (2) the two tumors are clonally related to each other. Given previous surgeries, we submitted schwannoma over the meningioma as Schwann cells are neural crest cell precursors. Schwannoma had the known heterozygous pathogenic variant (p.V333Sfs*15) but no second hit in *SDHA*. Instead, it had a double somatic hit in *NF1* possibly because of previous radiotherapy. This result does not remove the possibility of a secondary postzygotic event especially since nephroblastoma and GIST were so distant from each other. Dr van Dongen and colleagues described an equally rare and unusual clinical scenario in 2004 of a coexisting clonally unrelated non-Hodgkin lymphoma and a clonally unrelated myelomonocytic leukemia cutis in an 11-year-old girl with precursor-B-ALL.^10^

Tumor profiling in GISTs is not performed routinely for patients outside of testing for *KIT* and *PDGFRA*. Benefits of broader testing are unclear today as we only have a few tyrosine kinase inhibitor (TKI) options. Further characterization of somatic alterations in SDH-deficient GISTs is critically needed to advance development of new kinase inhibitors and to identify whether patients would benefit from chemotherapy agents such as fibroblast growth factor receptor inhibitors.^11^ Furthermore, there is insufficient knowledge on bioavailability of TKIs for fit patients who have significant chronic kidney disease. Our patient was started at half dose of sunitinib given that this chemotherapy was not studied in patients with GISTs with creatinine >2 upper limits of normal, his creatinine clearance was below 30 mL/min, and active metabolite of sunitinib can be found in urine of healthy volunteers up to 110 hours after administration. Our patient was not receiving dialysis during treatment with sunitinib, and he produced <750 cc of urine daily. Many cancer centers do not offer pharmacogenetic testing outside of indications such as dihydropyrimidine dehydrogenase for fluorouracil toxicity. More research is needed on bioavailability of TKIs for dose adjustment, measure response and resistance, and anticipate next line of therapy especially when only a few chemotherapies exist to treat patients with unresectable GISTs. Finally, germline genetic testing is recommended for every patient with SDH-deficient GIST and should be expanded to (1) every patient with GIST, (2) patients with cancer history especially if paraganglioma or adrenal lesions are present on imaging, (3) cancer family history of renal cell cancers or pheochromocytoma, and (4) somatic mutations in *SDH(x)*. More studies on tumors, patients, and families are needed to inform whether *SDHA* pathogenic variants are associated with a specific cancer risk profile compared with *SDHB*, *SDHD*, or *SDHC*. Screening recommendations can be tailored to family history but overall stay the same for all pathogenic variants in *SDH(x)*. Screening is lifelong, on the basis of review of systems, physical examination, plasma-free metanephrines levels, and regular cross-sectional skull base-to-pelvis MRI imaging. Metanephrines are repeated before a planned surgery and/or during pregnancy given risk of labile blood pressure and pre-eclampsia. Upper endoscopy is added for persistent GI symptoms or unexplained iron deficiency anemia. Recommending ad hoc upper endoscopy may be inadequate if a subset of patients with *SDHA* pathogenic variants are at increased risks of SDH-deficient GISTs compared with others.

In conclusion, our patient presented with multiple cancers known to be associated with familial PGL/PCC. Paired somatic and germline genetic testing helped identify a unifying diagnosis and characterize a clonal relationship between two of his three cancers. Our team is hoping to learn more from the patient-derived xenograft model. He was grateful to have a diagnosis and to be able to help advance knowledge on *SDHA*.
